# Volumineux œdème vulvaire comme mode de révélation d'une grossesse môlaire: à propos d'un cas

**DOI:** 10.11604/pamj.2019.33.23.18937

**Published:** 2019-05-14

**Authors:** Mamadou Ibrahima Kampo, Seydou Sogoba

**Affiliations:** 1 Hôpital de Tombouctou, Tombouctou, Mali

**Keywords:** Grossesse molaire, pré-éclampsie, tuméfaction vulvaire, Molar pregnancy, preeclampsia, vulvar swelling

## Abstract

Molar pregnancy is a benign trophoblastic disease usually discovered in patients with metrorrhagia and early miscarriage. We here report a case of molar pregnancy associated with severe preeclampsia in a 18-year-old primiparous woman presenting with early onset of vulvar swelling occurring in a three-day period. The patient reported metrorrhagias, headaches, vulvar pruritus lasting for a week. Physical examination on admission showed severe hypertension 150/110 mmHg with positive dipstick proteinuria +2 and discreet edema on the lower limbs. Fundal height was 20cm. Gynecological examination showed voluminous vulvar swelling with positive Godet sign sensitive to palpation (A, B). Blood test showed Rh positive blood type A, haemoglobin level 8.2 g/dl and positive qualitative HCG. Ultrasound revealed the absence of an embryo, “honeycomb-like multiple cysts and bilateral ovarian lutein cysts. No differential diagnosys, such as allergic reaction or vulvar infectious due to herpes, was suspected. Electric vacuum aspiration was performed and trophoblastic vescicular debris were collected. Anatomopathological examination helped to confirm the diagnosis of molar pregnancy. Immediately after, the patient was transfused with a unit of blood and received antihypertensive treatment. Clinical monitoring, laboratory tests and routine ultrasound were performed with favorable outcome and regression of the vulvar edema.

## Image en médecine

La grossesse môlaire est une maladie trophoblastique bénigne découverte habituellement dans un contexte de métrorragie et de fausse couche précoce. Nous rapportons un cas de grossesse môlaire associée à une pré-éclampsie sévère chez une primigeste de 18 ans consultant pour une tuméfaction vulvaire d'installation rapide en trois jours. La patiente rapporte des métrorragies, des céphalées, un prurit vulvaire depuis une semaine. L'examen d'admission révèle une hypertension artérielle sévère à 150/110 mmHg avec une protéinurie positive à 2 croix à la bandelette urinaire et un discret œdème des membres inférieurs. La hauteur utérine était de 20cm. L'examen gynécologique était marqué par une volumineuse tuméfaction vulvaire prenant le godet, sensible à la palpation (A, B). Le bilan sanguin a montré un groupe sanguin A rhésus positif, un taux d'hémoglobine à 8,2 g/dl et des βHCG qualitatifs positifs. L'échographie note l'absence d'embryon, un aspect multikystique en « nid d'abeilles » et des kystes lutéiniques ovariens bilatéraux. Nous n'avons pas retrouvé d'argument en faveur des diagnostiques différentiels tels une réaction allergique ou une cause infectieuse herpétique vulvaire. Une aspiration électrique intra-utérine ramène des débris trophoblastiques vésiculaires. L'examen anathomo-pathologique a confirmé le diagnostic de grossesse molaire. Une transfusion d'une unité de sang total est faite dans les suites immédiates ainsi qu'un traitement anti-hypertenseur. Une surveillance clinique, biologique et échographique est instaurée avec des suites simples et une régression de l'œdème vulvaire.

**Figure 1:**
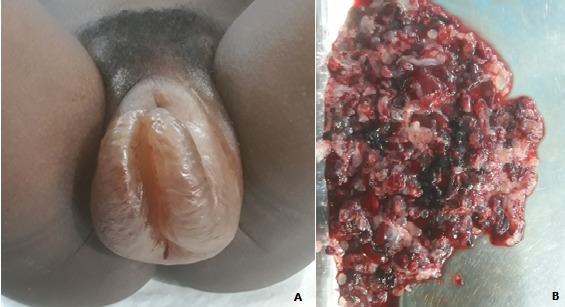
A) volumineuse tuméfaction vulvaire avec métrorragie minime révélant une grossesse môlaire; B) aspect macroscopique des débris vésiculaires du produit d'aspiration intra-utérine

